# Medical Recognition

**Published:** 1887-08

**Authors:** 


### Editorial.

### MEDICAL RECOGNITION.

In the last number of this journal we reviewed the action of the American Medical Association in passing the following resolution:

*Resolved,* That the regular graduates of such dental and oral schools and colleges as require of their students a standard of preliminary or general education and a term of professional study equal to the best class of the medical colleges of this country, and embrace in their curriculum all the fundamental branches of medicine, differing chiefly by substituting practical and clinical instruction in dental and oral medicine and surgery, in place of practical and clinical instruction in general medicine and surgery, be recognized as members of the regular profession of medicine, and eligible to membership in this Association on the same conditions and subject to the same regulations as other members.

We expressed the opinion that if this were adhered to in good faith, it would tend greatly to elevate the standard of dental education, for the diplomas of such schools as complied with these reasonable requirements would alone be of value. It was within the power of medicine to stamp out our irregular colleges, and to put a stop to the granting of degrees to unqualified and ignorant persons. It was only necessary for medicine to strictly maintain the line where that resolution drew it, and in a short time a dental degree would be better evidence of proper professional training than would that of most medical schools. The resolution positively announced that the possession of such a diploma would entitle its holder to admission to the International Medical Congress, and this was a consummation devoutly to be wished. We were not certain that the authority of the American Medical Association to establish new degrees in medicine would be respected by all the world, or that its right to modify the rules of admission to a congress with which that association had really nothing to do would be acknowledged by foreign members, but it at least established a valuable precedent. We were well aware that the men who are leading members of the association are also influential members of the Executive Committee which is this year managing the Congress, and in any case we did not propose to look a gift horse in the mouth, but to take unhesitatingly the good which was offered us.

But a somewhat remarkable editorial article in the *Journal of The American Medical Association* of July 9th, causes us to enquire where this action is to cease, and whether all professional lines are to be wiped out. It should be remembered that the editor of that journal is the president of the Congress, and therefore his editorial utterances are an official expression, and have a specially authoritative significance. In speaking of the conditions for membership in the Congress, he says :

"In regard to the registration of educated dentists, about which there has been some question, it is sufficient to say that the same rule will be followed as governed at the London Congress of 1881. The establishment of a Section of oral and dental surgery is a full admission that it constitutes a part of the domain of general medicine and surgery, and that all who, by education and proper legal authority, practice in that special department, are 'members of the regular medical profession.' At the London Congress they registered with the common prefix 'Dr.,' as did a large portion of eminent members of the profession in other departments.

"At the Congress at Washington it will be proper for them to register with the title Dr., M. D., D. M. D. or D. D. S., according to the term of the authority conferring upon them the right to practice their profession."

If we can rightly comprehend this article, it goes altogether too far. It not only recognizes the graduates of such dental schools as come up to the requirements of the resolution of the American Medical Association, but it admits any one who lays claim, whether justly or unjustly, to the appellation "Doctor." It recognizes a title which has no professional significance in this country. Veterinary surgeons are "doctors," and every quack, every "root and yarb" pretender, is especially tenacious of his title of "doctor." If every one who chooses to come before the secretaries of the Congress and call himself a doctor is to be admitted without question, a diploma is of no use.

We were pleased that respectable graduates of reputable dental schools were to be recognized; but if all who by "proper legal authority practice in that (our) special department" are to be admitted, then are the rules which require members to be "of the regular medical profession" a mere farce. In the State of New York, for instance, the act regulating the practice of dentistry provides that any one who claimed to be a dentist at the time of the passage of the act should be entitled to registration, and this should be considered a qualification for practice under the law. A majority of these possessed no diploma, and many of them were the veriest charlatans. Yet they practice by "legal authority," and so may register as members of the Congress, and according to this interpretation are "members of the regular medical profession." The certificates of the examining boards of different States are "legal authority" for practice, and hence make a man a "member of the regular medical profession" and entitle him to registration in the Congress. Verily the respected editor of the journal of the A. M. A. will make the whole affair ridiculous, if the obvious interpretation of his article is to be accepted.

It seems to us that it is time to call a halt in this matter. A recognition without discrimination is valueless, and the good which we had hoped for from the original resolution of the A. M. A. may be turned to evil, for instead of putting a premium upon a thorough dental education, this interpretation of it destroys the value of a diploma by placing its possessor upon the same level with the uneducated. We desired that the door of admission to the Congress should be opened a little wider, until it would allow the entrance of the educated dentist, but we did not ask that the walls should be leveled with the ground. We asked recognition for the qualified dentist, but we did not seek to have all lines of demarkation wiped out. We sought fellowship for educated men, and not that under pretence of granting our petition every one who pretends to practice dentistry should be given the same standing. If this is to be the result of the late action, we can only say that it will tend to degrade rather than to elevate dentistry, for our best men have been laboring to build up a wall separating the qualified from the unqualified, and this action would again break down the barrier and render nugatory all past efforts.

We sincerely hope that the editorial in question was but a careless piece of writing rather than the expression of a settled purpose, and that mere legality will not be the rule of admission to the Congress.

